# CDW-Based Geopolymers: Pro and Cons of Using Unselected Waste

**DOI:** 10.3390/polym17050570

**Published:** 2025-02-21

**Authors:** Ilaria Capasso, Gigliola D’Angelo, Mercedes del Río Merino, Assunta Campanile, Domenico Caputo, Barbara Liguori

**Affiliations:** 1 Department of Engineering and Geology, University of Chieti-Pescara “G d’Annunzio”, Viale Pindaro 42, 65122 Pescara, Italy; ilaria.capasso@unich.it; 2 DICEA—Department of Civil, Building and Environmental Engineering, University of Naples Federico II, P.le Tecchio 80, 80126 Naples, Italy; gigliola.dangelo@unina.it; 3 Grupo de Investigación TEMA, Escuela Técnica Superior de Edificación, Universidad Politécnica de Madrid, 28040 Madrid, Spain; mercedes.delrio@upm.es; 4 ACLabs—Applied Chemistry Labs, Department of Chemical, Materials and Industrial Production Engineering, University of Naples Federico II, P.le Tecchio 80, 80125 Naples, Italy; assunta.campanile@unina.it (A.C.); domenico.caputo@unina.it (D.C.)

**Keywords:** unselected demolition waste, historical buildings, geopolymer, mortars, circular economy

## Abstract

Geopolymer technology is considered a strategic alternative for recycling construction and demolition waste (CDW) and to produce new construction products which meet the requirements of environmental and energy sustainability. The separation and management of CDW fractions is still a technological complex process and, even if large-scale separation technology is quite common, the necessity to perform this treatment may reduce the environmental and economic benefits of CDW reuse. So, a very promising option is represented by the manufacturing of geopolymers using unseparated CDW. In this aim, waste deriving from cement-based mortars, bricks and natural stones have been selected and widely characterized from a mineralogical, chemical and morphological point of view. Then, geopolymer mortars were produced using several amounts of either a single fraction or a mixture of the selected waste. The chemical, physical, mechanical, and microstructural characterization of the geopolymer-produced mortars was carried out to assess how the combination and different quantities of the mixed CDW affected the final properties. In particular, geopolymeric mortars produced from the unselected CDW showed higher mechanical properties, despite the lower apparent density, when compared to geopolymeric mortars produced from single fractions of CDW. The improvement of mechanical features seems to be not affected by the waste amount used, providing encouraging findings to promote the actual use of unseparated CDW with the resulting enhancement of environmental and economic benefits.

## 1. Introduction

The low durability of the traditional buildings, mainly due to their high vulnerability to environmental factors such as moisture, temperature fluctuations, and chemical exposure which can accelerate their deterioration, the rapid growth of global urban areas and the infrastructure development combined with the requirement of building renovation, generate intensive demolition activities. The major consequence of the continuous increase in demolition activities mainly results in the production of large amounts of mixed waste containing concrete, bricks, glass, tiles, metals, and plastics, which are defined as construction and demolition waste (CDW) [1,2]. Global construction and demolition waste (CDW) production is estimated to reach around 10 billion tons every year with approximately 800 million tons produced solely by the European Union [3].

In recent years, there has been a growing interest in CDW, which started to be considered as a huge and still undervalued resource [4,5,6]. Today, an important step forward has been taken in Italy toward the changes promoted by the ecological transition, thanks to Ministerial Decree no. 152 of 27 September 2022, that identifies and defines the criteria by which inert materials from construction and demolition activities will no longer be considered as waste but rather as recovered aggregates. As a result, the early perspective of recycling as merely the recovery of significant amounts of waste without any added value has evolved into a stronger emphasis on the innovative repurposing of waste, which is progressively aligning with the principles of a circular economy. A promising approach is based on reusing large quantities of construction and demolition waste in the production of geopolymeric mortars as sustainable alternative to traditional building materials [4,5,7,8,9,10,11,12]. Geopolymers are alkali-activated aluminosilicates [13], which have been extensively studied due to their ability to be easily produced at low curing temperatures [14] and to be obtained from waste precursors like industrial, agricultural, mining, construction and demolition, quarrying, electronic, and municipal solid wastes [15,16,17,18,19,20,21,22,23,24,25,26]. Thus, geopolymers are very cost-effective and sustainable [27,28,29]. Furthermore, the use of waste as raw material for geopolymers solves the problem of finding available disposal sites and also prevents hazardous materials contaminating the environment [30,31]. In addition, compared to Portland cement substitutes, geopolymers show higher performance in terms of mechanical strength, which can reach values of compressive strengths up to 70 MPa, and durability, especially considering their ability to withstand effects of aggressive environments [32]. In particular, geopolymers exhibit excellent resistance to sulfates, chlorides and acid attacks, which often damage traditional cement, and also to acidic and mechanical stresses at high temperatures, preserving mechanical properties about 30 MPa after exposure to high temperature up to 800 °C [32,33].

The existing historical building heritage is widespread and heterogeneous, and it is composed of different types of natural or artificial stones and materials [34,35,36,37]: looking at the European building heritage, tuff, marble and brick are the most used building materials. In addition, in recent decades, cement has been extensive used in plaster restoration.

As reported by Tan et al. and Papamichael et al. [38,39], the recent literature was mainly focused on alkali activation of the single fraction separated from CDW. The separation and management of CDW fractions is still a technological complex process; therefore, to enhance the environmental and economic benefits of this practice, a great challenge is represented by using unselected wastes [38]. Few papers deal with the joint utilization of CDW [40,41] in geopolymerization processes that demonstrate an interesting synergy related to the combination of CDW in geopolymer production.

Based on the promising results obtained for brick and tuff waste-based geocomposites in a previous paper [42], in the present paper, the authors aim at evaluating the feasibility of unselected construction and demolition waste coming from historical buildings as an aluminosilicate source and/or aggregate in geopolymer-based mortars production. Tuffs, bricks and cement-based mortars waste have been studied, and their mineralogical, chemical and morphological properties have been preliminary evaluated to assess their suitability as a geopolymer precursor.

Then, several geopolymer mortars were produced using either a single fraction or a mixture of the waste. Chemical, physical, mechanical and microstructural characterization were performed to understand the effect of the combination of the CDW on geopolymer mortars’ performances to promote their possible future applications as innovative and sustainable materials in building activities.

## 2. Materials and Methods

### 2.1. Raw Materials and Preparation of the Geocomposites

Construction and demolition waste of different origins have been employed to produce geopolymeric mortars, whether as geopolymeric precursors or as natural aggregates replacement (see Supplemental Figure S1):

- Waste 1 (W1) is coming from the demolition of cement-based plasters on the façade of an historical building (Supplemental Figure S1a);
- Waste 2 (W2) is brick waste (Supplemental Figure S1b);
- Waste 3 (W3) is natural stones waste, composed by a mixture of two typical Italian tuffs, Neapolitan Yellow Tuff (NYT) and Viterbo Red Tuff (VRT) (Supplemental Figure S1c).

For geopolymer production, the waste fractions with particle size between 0.3 and 4 mm are used as aggregates, whereas the fraction with particle size lower than 0.3 mm is used as a geopolymeric precursor. In addition, also fly ashes (FAs), from power generation plants, have been used as reactive addition to produce the waste-based geopolymer specimens. A set of mixed geopolymer (MW) was manufactured with all types of the above selected waste both in the binder and aggregate phase. 

The alkaline activator was obtained as follows: a sodium silicate solution (SS) (Na_2_O 8.15%, SiO_2_ 27.40%), supplied by Prochin Italia S.r.L. (Caserta, Italy), was mixed with a 10 M sodium hydroxide solution (N) prepared by dissolving NaOH pellets (NaOH 98%, J.T. Baker) in bi-distilled water. The weight ratio SS/N/binder was 1:1:3 and the activator/binder ratio was fixed at 0.66 for all the mixtures based on the optimization of experimental parameters deriving from previous studies [43,44].

Geopolymeric mortars were obtained by mixing and homogenizing the powdered materials and then adding the activator solution to the dry mixture. Then, the aggregates were added in a weight to binder ratio of 0.5 and, after 2–3 min of mixing, slurries were poured into appropriate molds.

Three different sample geometries were produced in accordance with the specific requirements of the European standards for physical and mechanical characterization (see Section 2.4 and Section 2.5):

- Cylindrical (diameter 45 mm; height 50 mm);
- Cubic (side 50 mm);
- Prismatic (40 × 40 × 160 mm^3^).

After pouring, the samples were first vibrated for about 60 s to remove air bubbles entrapped in the mortars and then stored in sealed vessels to ensure 100% relative humidity and cured at 60 °C for 72 h. At the end of the curing period, all the specimens were removed from the molds and stored at room temperature. Three specimens for each set of geopolymeric mortar were prepared. The composition and labels of all the mixtures are summarized in Table 1.

### 2.2. Characterization of the Raw Materials

To assess the mineralogical and chemical composition of the selected waste, XRD (using a Malvern Panalytical, Malvern, United Kingdom, X’Pert Pro diffractometer equipped with PixCel 1D detector, operative conditions: CuKα1/Kα2 radiation, 40 kV, 40 mA, 2θ range from 5 to 60°, step size 0.0131 °2θ, counting time 40 s per step) and EDS (Oxford CS-2280, Oxford Instrument, High Wycombe, UK) analyses were performed on powdered samples. Furthermore, a morphological investigation was carried out with scanning electron microscopy (Tescan Vega II XM, Tescan, Brno—Kohoutovice, Czech Republic).

### 2.3. Chemical and Morphological Characterization of the Geopolymeric Mortars

Further information about the degree of geopolymerization and about the influence of the CDW addition on the chemical and morphological features of the samples produced was deduced by means of FTIR and SEM analyses. The chemical characterization has been performed by FTIR spectroscopy at room temperature by using a Spectra 3000 (Perkin Helmer, Waltham, MA, USA) in ATR mode and selecting a wavenumber resolution of 4 cm^−1^ for 32 scans from 4000 to 600 cm^−1^. Furthermore, the fractured surfaces of geopolymer samples were coated with gold and observed by a Tescan Vega II XMU microscope to evaluate the main microstructural features.

### 2.4. Physical Characterization of the Geopolymeric Mortars

The open porosity and the water absorption of the geopolymeric mortars were evaluated in accordance with UNI 11060:2003 [45]. The specimens were dried at 60 ± 5 °C until they reached a constant mass (*M*_1_, g) and after they were submerged in water at room temperature within an evacuation vessel where the pressure was reduced to approximately 20 mmHg and maintained for 2 h. Then, the pressure was restored to atmospheric level, and the samples were weighed and submerged in water (hydrostatic weighing, *M*_2_, g). Finally, after being gently wiped with a damp cloth, their water-saturated mass (*M*_3_, g) was determined. Each test was conducted in triplicate, and the results are presented as average values. Water absorption (*WA*%), open porosity (*OP*%) and apparent density (*ρ_app_*, g mL^−1^) were expressed as follows:

(1) $$WA\%=\frac{M_{3}-M_{1}}{M_{1}}\times 100$$

(2) $$OP\%=\frac{M_{3}-M_{1}}{M_{3}-M_{2}}\times 100$$

(3) $$\rho_{app}=\frac{M_{1}}{V_{app}}$$

The apparent volume (*V_app_*, mL) in Equation (3) was calculated as $M_{2}/\rho_{water}$.

Furthermore, to verify the pores interconnection, a capillarity test was performed in accordance with the European standard UNI EN 15801 [46]. The tests were conducted in triplicate on cylindrical samples, so it was possible to determine the average values of water absorbed by capillarity (Q, kg m^−2^) and capillary absorption coefficient (CA, g cm^−2^ s^−1/2^). According to the standard, the capillary absorption coefficient CA was calculated as the slope of the straight line in the first 30 min of the capillarity test, considering that for short times, the relationship between adsorbed water (Q) and the square root of time is quite linear.

### 2.5. Mechanical Characterization of the Geopolymeric Mortars

The mechanical features were measured according to the European standard UNI EN 196-1:2016 [47]. After 28 days of curing, three prismatic specimens (40 × 40 × 160 mm) were tested in a 3-point flexure test with an Ibertest testing machine (Ibertest, Madrid, Spain). Then a compression test was performed on two sample pieces resulting from the flexure test.

The surface hardness was measured by applying a fixed force to the top surface of the prismatic sample in accordance with European standard UNI EN 13279-2:2014 [48]. 

## 3. Results and Discussion

### 3.1. Characterization of Raw Materials

**Figure 1:**
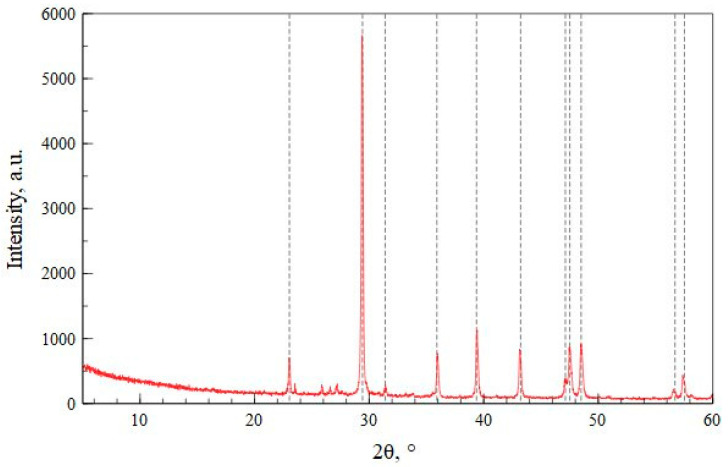
XRD spectrum of W1 (CaCO_3_ REF. CODE 01-078-4614, dotted lines).

Crystalline structures of the geopolymer precursors were reported in Capasso et al. [42]. Figure 1 shows the XRD spectrum of W1, which reveals the presence of calcite as the main crystalline phase.

Table 2 shows the chemical compositions and mineralogical phases present in all the selected waste. Excluding the oxygen content, silicon (Si) is the most abundant phase in all wastes; only W1 is an exception in which calcium (Ca) is the most abundant phase. 

The FA showed the typical morphology of ashes characterized by small spherical particles (see Supplemental Figure S2), which are responsible for their high reactivity in promoting the geopolymerization process [44]. W2 particles have irregular and angular-shape morphology [49] with a wide particle size distribution ranging from 1 to 30 microns. In the case of W1, as confirmed by chemical and mineralogical analysis, the presence of rhombohedral crystals is observed, showing a typical layered structure of CaCO_3_ and calcite [50]. The presence of zeolitic phases in tuff waste, already showed by XRD data, is further confirmed in SEM images (Supplemental Figure S3). Accordingly, different morphologies such as acicular clusters consisting of thin prismatic crystals for phillipsite, and pseudo-cubic crystals for chabazite, can be clearly distinguished [51,52,53].

### 3.2. Chemical and Morphological Characterization of the Geopolymeric Mortars

Successful geopolymerization can be assessed qualitatively by completely immersing the geocomposites in bi-distilled water for 1 day at room temperature. If the samples are unbroken and undissolved after the immersion period, geopolymerization has occurred correctly [23,54]. All geocomposites have passed the geopolymerization test (see Supplemental Figure S4).

**Figure 2:**
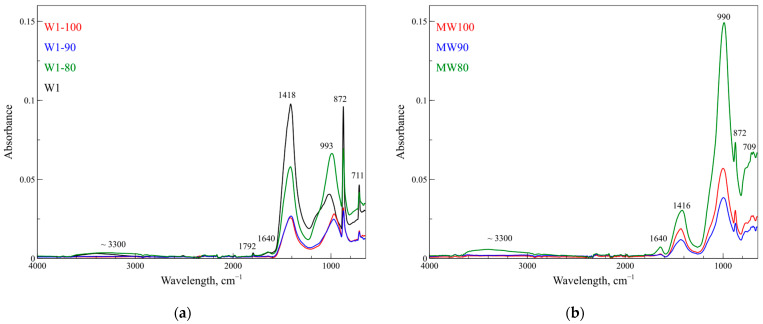
FT-IR spectra of W1- (**a**) and MW-based (**b**) geopolymers.

Figure 2 shows FT-IR spectra of all geocomposites. In particular, the bands around 700, 870 and 990 cm^−1^ are attributed to the asymmetric stretching of Si-O-Si and Si-O-Al bonds. The band around 1410 cm^−1^ is attributed to the O-C-O symmetric stretching of the carbonate, and the less intense band around 1640 cm^−1^ is attributed to the adsorbed water vibrational bending mode [55,56,57]. The peak shift and intensity variation compared with the geopolymer precursor (W1, black line in Figure 2a) confirm the successful geopolymerization in all samples.

**Figure 3:**
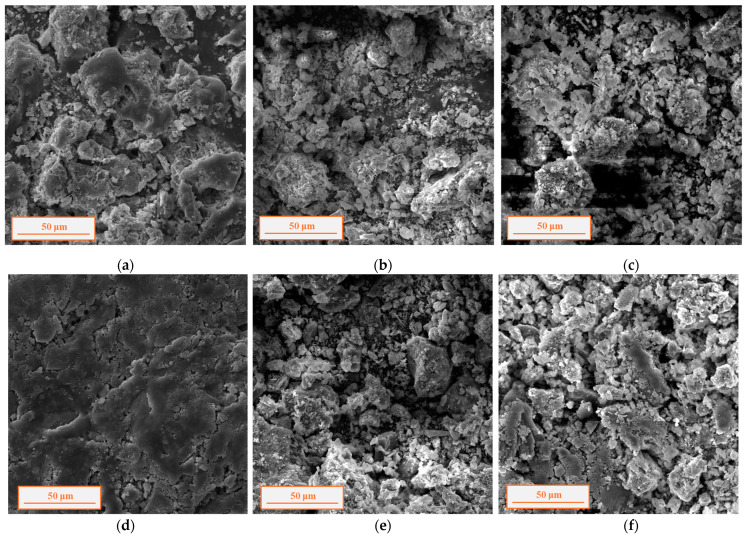
SEM analyses (magnification 1200×) of fragments of W1 samples: (**a**) W1-80, (**b**) W1-90, and (**c**) W1-100 and of MW samples (**d**) MW80, (**e**) MW90 and (**f**) MW100.

Geopolymerization is also confirmed by SEM analysis (Figure 3): greater compactness is highlighted in geopolymers with the highest percentage of FA, W1-80 (Figure 3a) and MW-80 (Figure 3d).

### 3.3. Physical Characterization of the Geopolymeric Mortars

The effects of the addition of different percentages of waste on some relevant physical characteristics of geopolymer composites (density, water adsorption, water-accessible porosity, and water adsorption by capillarity) were evaluated, and the experimental results are shown in Table 3 as mean values with relative standard deviation. In the case of MW-based geopolymers, apparent density decreases as the percentage of waste used increases, consequently water-accessible porosity (Open Porosity) and water absorption increase, by about 10%. The same trend is also confirmed by capillary water absorption tests (data reported in Supplemental Figure S5).

A similar trend can be identified also for W1-based geopolymers. In fact, W1-80 has the highest apparent density, while W1-90 and W1-100 show very similar density values. The higher compactness of W1-80 is confirmed by the lower open porosity and water absorption; on the contrary, after increasing the percentage of waste from 80% to 100%, both increase by about 10%.

### 3.4. Mechanical Characterization of the Geopolymeric Mortars

The highest values in terms of compressive strength are obtained with geopolymers W1-80 and MW80, in which the amount of fly ash is higher. These improved mechanical features can be associated with the higher degree of geopolymerization due to the presence of fly ash, as previously confirmed both by the increase in peak intensity in the FT-IR spectra and from porosity values.

MW-based geopolymers turn out to be more mechanically resistant than W1-based geopolymers. Most likely, the addition of other wastes (such as brick and tuff waste) enriches the chemical composition, in terms of silica and alumina amount, promoting the reactivity of precursors in forming stronger and more compact systems, as already confirmed by SEM analysis (Figure 3).

Surface hardness was measured in Shore D units, ranging from 0, for soft materials, to 100, for harder ones. All geocomposites showed hardness higher than 50%, and MW-based geopolymers followed the same trend of flexural and compressive strengths, resulting in them being harder than the W1-ones. Inspecting the relationship between the compressive strength and Shore D hardness of all the waste-based geopolymeric mortars (Supplemental Figure S6), it is possible to deduce that for the MW-based geopolymers, compressive strengths and hardness value are basically linearly correlated (correlation coefficient R^2^ = 0.98), while data related to W1-based samples do not follow a linear relationship but a second-degree polynomial function (R^2^ = 1). This means that, probably, experimental methods based on hardness testing to estimate the strength may not be applicable to W1-based geopolymeric mortars.

**Figure 4:**
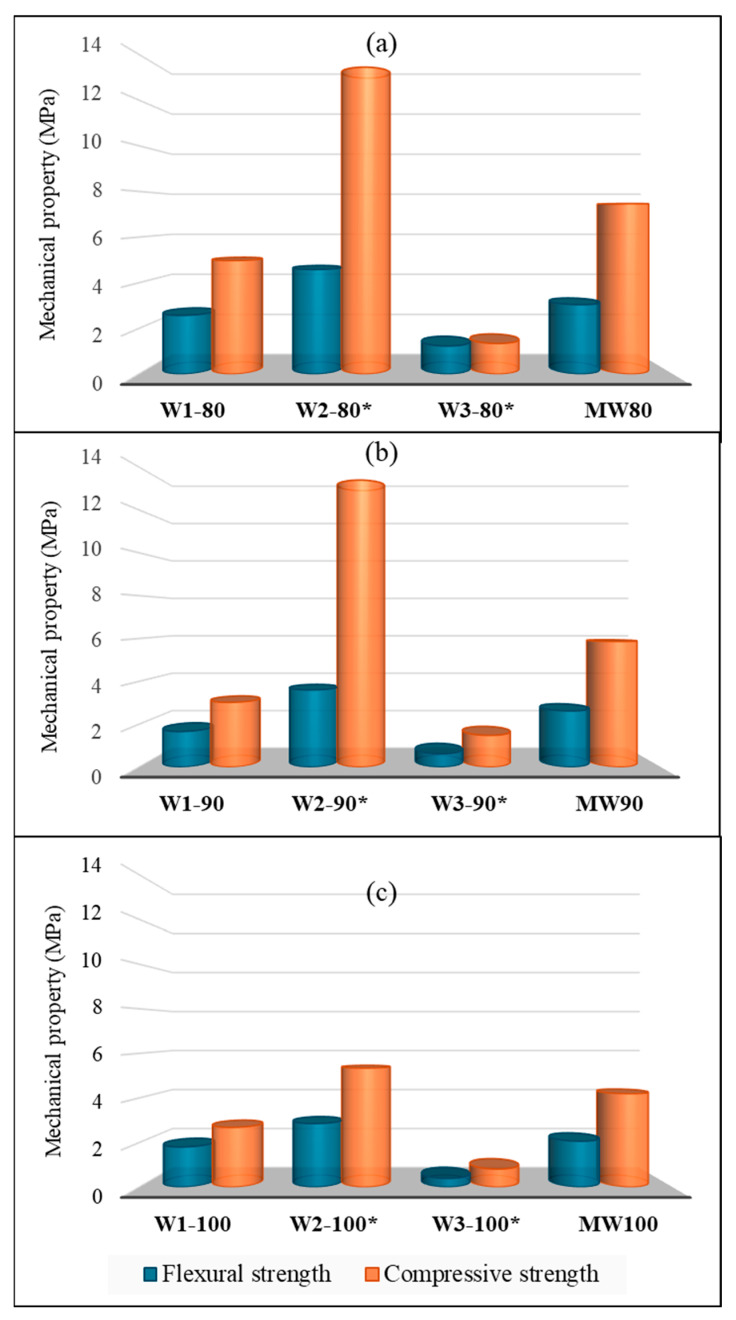
Comparison of the mechanical properties of several geopolymeric mortars produced from different waste and with different percentage of waste used: (**a**) 80%, (**b**) 90% and (**c**) 100%. * Data already reported in [42].

To validate the effectiveness of the combined use of different CDW, the experimental results obtained in this paper for the MW samples were compared with those of the geopolymeric mortars produced with each single waste used alone (W1, W2 and W3) but using the same procedure and set of experimental parameters (Figure 4). Experimental results related to W2 and W3-based geopolymeric composites have been widely discussed in our previous paper [42]. MW samples showed the best mechanical behavior both in terms of flexural and compressive strengths when compared to the geopolymeric mortars produced using cement-based waste and natural stone waste (W1 and W3) and regardless of the waste amount. Only brick waste-based geopolymers (W2) exhibited a better mechanical performance when used alone (see Figure 4), but this can be related to the chemical composition of brick waste and to their clay nature, which led to a higher silica and alumina reactivity in a high alkaline environment, promoting a more effective geopolymerization process [58]. The mechanical properties of MW-based samples led to the conclusion that the combined use of different waste revealed to be successful in the production of waste-based geopolymeric mortars. 

## 4. Conclusions

This paper studied the feasibility of using demolition waste of different nature, i.e., cement-based mortars, bricks and natural stones, in geopolymeric mortars production. Also, fly ashes have been used to improve the global reactivity of precursors. The effects of several waste amounts and waste combination on the performance of the resulting geopolymeric mortars were studied. 

The following remarks can be drawn based on the results:

1. All the waste selected showed suitable chemical and mineralogical features to be employed as a precursor and/or aggregate in geopolymer production, so the reaction of geopolymerization occurred successfully for all the waste-based geopolymeric mortars produced, as confirmed both by FT-IR and SEM analysis. More in detail, higher compactness can be detected in geopolymers with the presence also of fly ashes, which led to a higher degree of geopolymerization.
2. The addition of different percentages of waste differently affected the main relevant chemical–physical and morphological characteristics of geopolymeric mortars. In particular, a higher amount of waste promoted density reduction with a following porosity and water absorption increase.
3. Compared to the geopolymeric mortars produced using single fractions of both cement-based waste and natural stone waste, geopolymeric samples obtained from unselected waste exhibited the best mechanical behavior regardless of the waste amount. This is probably due to the simultaneous presence of different wastes (especially brick and tuff waste), which improved the chemical composition and increased the reactivity, producing stronger and more compact final geopolymeric composites. At the same time, the higher clayey nature of brick waste (W2) led to higher mechanical properties for brick waste-based geopolymers.

So, the waste-based geopolymers studied in this paper represent a promising alternative to traditional building materials due to the sustainability of the production process, the lower CO_2_ emissions and the possibility of introducing secondary raw materials, by preventing the exploitation of natural ones. Moreover, the feasibility of using unseparated demolition waste further optimizes both the environmental and economic impact of this kind of waste, reducing the necessity of their separation and sorting.

## Figures and Tables

**Table 1:** Geopolymeric mortar compositions.

Mixture	Binder				Aggregate		
	FA (% wt)	W1 (% wt)	W2 (% wt)	W3 (% wt)	W1 (% wt)	W2 (% wt)	W3 (% wt)
W1-80	20	80	/	/	100	/	/
W1-90	10	90	/	/	100	/	/
W1-100	/	100	/	/	100	/	/
MW80	20	26.66	26.66	26.66	33.33	33.33	33.33
MW90	10	30	30	30	33.33	33.33	33.33
MW100	/	33.33	33.33	33.33	33.33	33.33	33.33

**Table 2:** Chemical and mineralogical composition of waste.

		Waste				
		W1	W2	W3		FA
				NYT	VRT	
Chemical composition (wt% ± standard deviation)	Si	3.60 (±0.27)	25.43 (±0.13)	28.86 (±2.88)	27.66 (±0.03)	25.62 (±1.13)
	Al	1.68 (±0.23)	9.38 (±0.13)	8.74 (±0.70)	9.17 (±0.11)	14.53 (±2.14)
	O	56.74 (±0.86)	50.03 (±0.96)	49.28 (±1.97)	54.51 (±0.93)	51.65 (±2.02)
	Ca	32.29 (±1.10)	/	/	2.88 (±0.09)	4.28 (±0.10)
	K	/	1.79 (±0.20)	7.01 (±0.47)	6.03 (±0.64)	/
	Fe	1.59 (±0.13)	6.57 (±0.90)	4.09 (±0.27)	4.09 (±0.27)	2.59 (±0.01)
	Na	/	/	1.46 (±0.10)	/	/
Mineralogical composition		Calcite	Quartz, Hematite, Albite	Phillipsite-K, Analcime, Sanidine	Chabazite, Phillipsite-K, Analcime, Sanidine	Mullite, Quartz

**Table 3:** Main physical properties of geopolymeric mortars.

Sample	Apparent Density (g cm^−3^)	Open Porosity (%)	Water Absorption (%)	CA (mg cm^−2^ s^−1/2^)	Qmax (mg cm^−2^)
W1-80	1.56 ± 0.01	31.34 ± 0.53	20.07 ± 0.47	17.87 ± 1.26	1499.68 ± 15.59
W1-90	1.47 ± 0.01	34.35 ± 0.64	23.38 ± 0.49	19.35 ± 1.71	1530.00 ± 12.21
W1-100	1.45 ± 0.01	45.70 ± 0.23	31.47 ± 0.30	15.49 ± 0.40	1949.88 ± 7.48
MW80	1.52 ± 0.01	36.57 ± 0.39	24.00 ± 0.43	15.61 ± 1.97	1849.11 ± 16.57
MW90	1.49 ± 0.01	43.64 ± 0.13	29.22 ± 0.26	23.73 ± 0.57	1903.82 ± 6.22
MW100	1.38 ± 0.01	46.87 ± 0.06	33.96 ± 0.04	29.10 ± 1.38	1984.12 ± 4.40

## Data Availability

Data are available on request.

## Supplementary Materials

The following supporting information can be downloaded at https://www.mdpi.com/article/10.3390/polym17050570/s1, Figure S1: Demolition waste of (a) cement-based plasters (W1), (b) brick waste (W2) and (c) natural stones waste (W3), Figure S2: SEM analysis of FA (a), W2 (b) and W1 (c), Figure S3: SEM analysis of NYT (a) and VRT (b), Figure S4: Geopolymeric samples before (a), during (b), and after immersion (c), (d) in bi-distilled water for 24 h, Figure S5: Water absorbed by capillarity (Q, mg cm^−2^) as a function of time (t, √s) in W1 (a) and MW-based (b) geopolymers, Figure S6: Correlation between Shore D hardness and compressive strength for W1-based and MW-based geocomposites.

## Abbreviations

The following abbreviations are used in this manuscript:

CDW	construction and demolition waste (CDW)
NYT	Neapolitan Yellow Tuff
VRT	Viterbo Red Tuff
FA	fly ash
MW	mixed geopolymer
SS	sodium silicate solution
N	sodium hydroxide solution
CA	capillary absorption coefficient
Q	adsorbed water
