# Supplementary material for: CDW-Based Geopolymers: Pro and Cons of Using Unselected Waste
Source: Polymers (Basel). 2025 Feb 21;17(5):570. doi: 10.3390/polym17050570 (PMC11902754; doi:10.3390/polym17050570)
Supplement: Supplementary file 1 [file polymers-17-00570-s001.zip › polymers-3471010-supplementary.pdf]

CDW-Based Geopolymers: Pro and Cons of Using Unselected Waste

Ilaria Capasso[1], Gigliola D'Angelo[2], Mercedes del Rio Merino[3], Assunta Campanile[4], Domenico Caputo[4], Barbara Liguori[4,*]

[1] Department of Engineering and Geology, University of Chieti-Pescara "G d'Annunzio", Viale Pindaro 42, Pescara 65122, Italy, ilaria.capasso@unich.it
[2] DICEA - University of Naples Federico II, P.le Tecchio 80, Naples 80126, Italy, gigliola.dangelo@unina.it
[3] Universidad Politécnica de Madrid, Escuela Técnica Superior de Edificación. Grupo de investigación TEMA, Madrid, 28040, Spain, mercedes.delrio@upm.es
[4] ACLabs - Applied Chemistry Labs, Department of Chemical, Materials and Industrial Production Engineering, University of Naples Federico II, P.le Tecchio 80, Naples 80125, Italy, assunta.campanile@unina.it (A.C.); domenico.caputo@unina.it (D.C.)
* Correspondence: barbara.liguori@unina.it

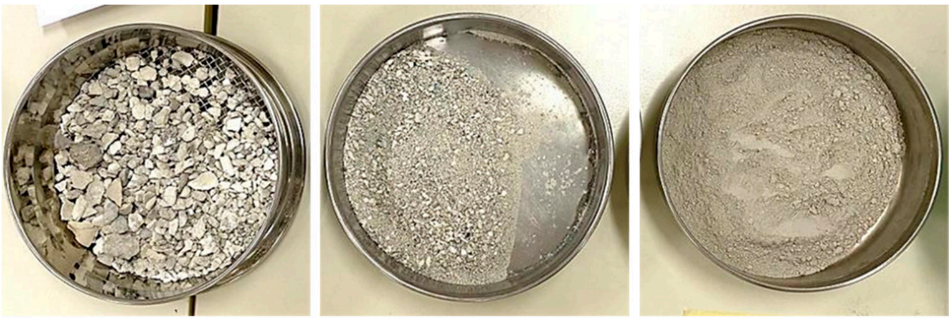

r > 4 mm   4 mm > r > 0.3 mm   r < 0.3 mm

(a)

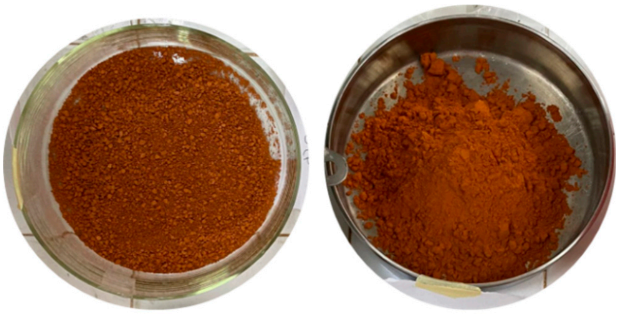

4 mm > r > 0.3 mm   r < 0.3 mm

(b)

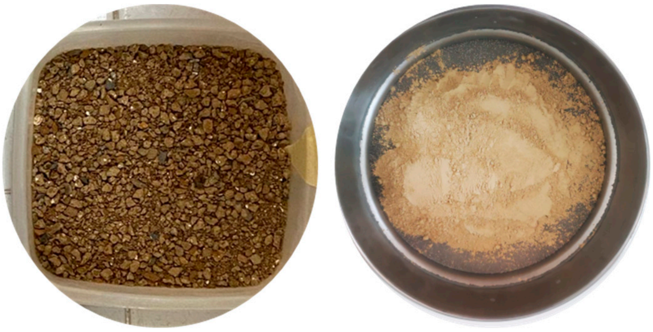

4 mm > r > 0.3 mm   r < 0.3 mm

(c)

Figure S1. Demolition waste of (a) cement-based plasters (W1), (b) brick waste (W2) and (c) natural stones waste (W3).

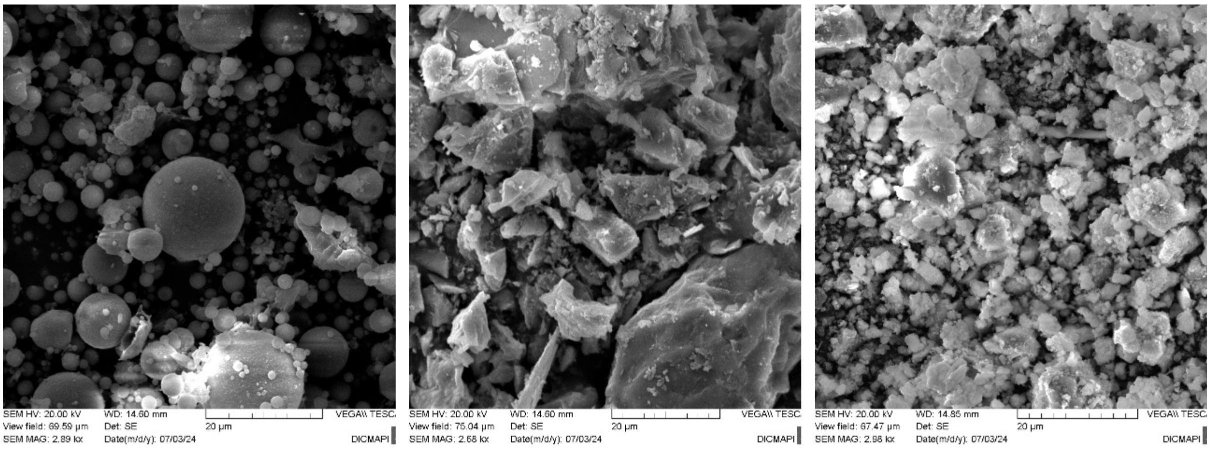

(a) (b) (c)

Figure S2. SEM analysis of FA (a), W2 (b), and W1 (c).

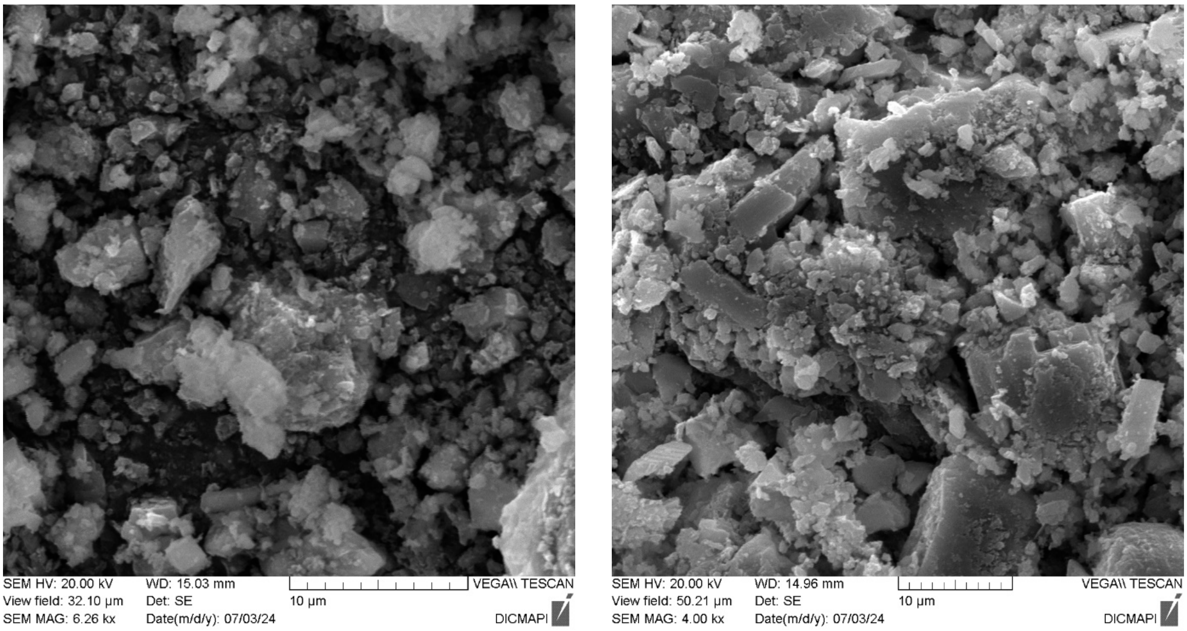

(a) (b)

Figure S3. SEM analysis of NYT (a) and VRT (b).

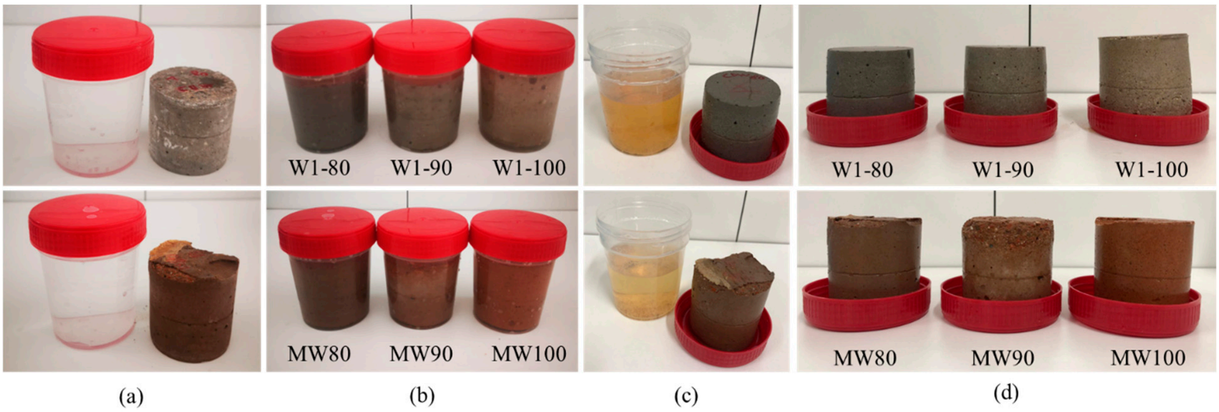

Figure S4. Geopolymeric samples before (a), during (b), and after immersion (c) (d) in bi-distilled water for 24 h.

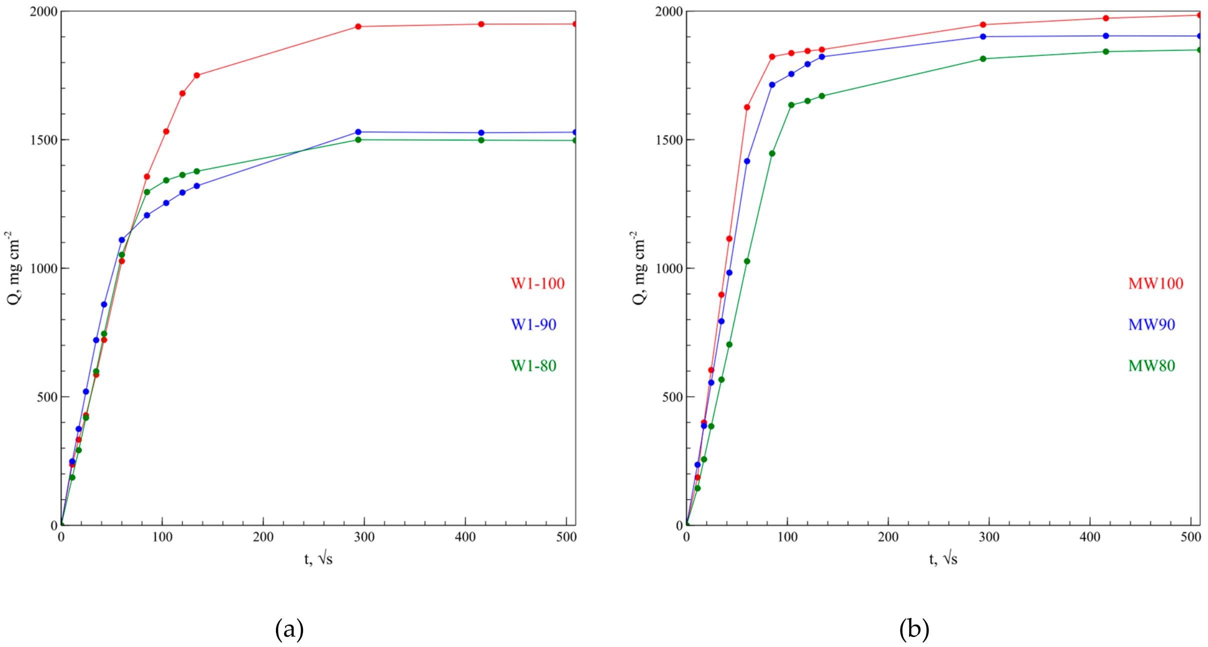

Figure S5. Water absorbed by capillarity (Q, mg cm^{-2}) as a function of time (t, √s) in W1- (a) and MW-based (b) geopolymers.

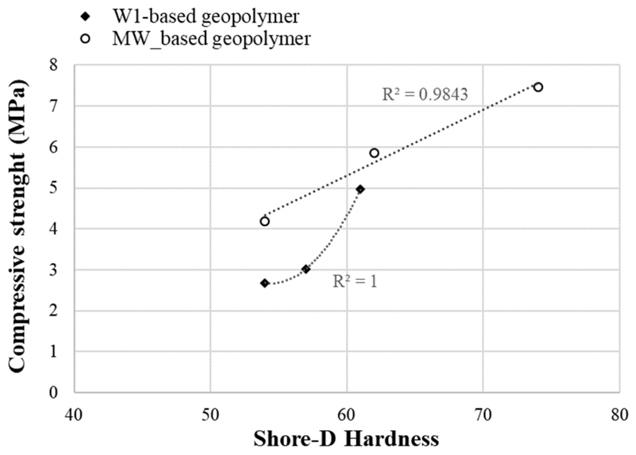

Figure S6. Correlation between Shore D hardness and compressive strength for W1-based and MW-based geocomposites.
